# Social inequalities in medical appointment cancellations and reschedulings at the onset of the COVID-19 epidemic in France

**DOI:** 10.1093/eurpub/ckae101

**Published:** 2024-06-27

**Authors:** Jeanna-eve Pousson, Florence Jusot, Léna Silberzan, Nathalie Bajos, EpiCov Study Group, Guillaume Bagein, Emilie Counil, Florence Jusot, Nathalie Lydie, Laurence Meyer, Philippe Raynaud, Alexandra Rouquette, Ariane Pailhé, Delphine Rahib, Patrick Sillard, Alexis Spire

**Affiliations:** INSERM-IRIS (UMR8156 - U997), INSERM, Aubervilliers, France; Health Department, Université Paris Dauphine, 8 Boulevard Lannes, Paris, 75116, France; INSERM-IRIS (UMR8156 - U997), INSERM, Aubervilliers, France; INSERM-IRIS (UMR8156 - U997), INSERM, Aubervilliers, France; IRIS, EHESS, 25 Rue du Pilier, Aubervilliers, 93322, France

## Abstract

Inconsistent results are found regarding social inequalities related to healthcare appointment cancellations during the COVID-19 crisis. Whether rescheduling was associated with social status is unknown. By studying both cancellations and rescheduling, we comprehensively describe which social groups were affected by care disruption. First follow-up of a random population-based cohort was used, including 95 118 people aged 18 or older at baseline and who live in France. Poisson and multinomial regressions were used to study social factors associated with experiencing both medical appointment cancellation by health professionals during the first COVID-19 lockdown, and rescheduling within six months. Among all individuals (including those without scheduled appointment), 21.1% reported cancellations initiated by healthcare professionals. Women, the richest, and those with a chronic disease were the most affected by these cancellations. Although 78.1% who had their appointment cancelled obtained a new appointment within six months, 6.6% failed to reschedule and 15.2% did not want to reschedule. While the oldest were more likely to reschedule, regardless of their health status, the poorest and those with multiple chronic diseases were less likely to do so. Difficulties in rescheduling revealed certain social groups were ultimately more penalized by the restriction of access to care during the first wave of the COVID-19 pandemic. Given that the poorest people, a social group that is in poorer health condition compared to other groups, were the most affected, our results raise questions about the ability of the healthcare system to reduce social health inequalities during a major health crisis.

## Introduction

To limit the spread of COVID-19 early in the epidemic, a first lockdown was implemented in France for two months starting on 17 March 2020. To concentrate the resources of the healthcare system into treating COVID-19 patients and to avoid the risk of virus transmission to healthcare workers and patients, medical care was restricted to the most urgent procedures and treatments, which led to drastic healthcare rationing. Many hospital and outpatient appointments were cancelled or postponed by healthcare professionals or by patients themselves, some of the patients being afraid of being exposed to the virus. This major restriction on access to the healthcare system is likely to have affected people differently depending on their pattern of medical use prior to the epidemic. Despite the overall high performance of the French healthcare system and the fact that 95% of people have a private supplementary health insurance, social inequalities in healthcare utilization exist [1–4]. In particular, pro-rich inequalities are important for access to specialist and prevention care [4]. As the activity of specialist physicians decreased by 60%, against 30% for general practitioners (GPs) during the first lockdown in France [5], upper social classes may have experienced more cancellations by healthcare professionals than lower social classes. Cancellations may have been more substantial for certain types of care more used by the poorest such as hospital care or care delivered by public sector doctors. On the other hand, people with low socioeconomic status are more likely to forgo care or use unscheduled care options, including visit to emergency departments [6, 7]. Gender inequalities also exist, women visiting healthcare professionals more often than men, partly due to their gynaecological follow-up [8]. Ethno-racial minorities, in particular, the first-generation immigrants, are likely to face barriers in accessing health care, including lack of knowledge about the French healthcare system or discrimination [9]. In all, it can be assumed that cancellations initiated by healthcare professionals most affected people who have a greater use of healthcare services, i.e. older adults, individuals with multimorbidity, women, and rich people.

Previous studies in the USA, UK, Canada, and European countries have investigated which populations reported the most healthcare cancellations in 2020. Cancellation rates between 17% and 48% have been reported for the general population [10–15]. While women and people with chronic diseases were more likely to report healthcare cancellations, conflicting results were found regarding age, ethnicity, and socioeconomic position. However, none of the studies made a distinction between cancellations initiated by individuals and those initiated by healthcare professionals, nor did they study whether it was possible to reschedule these cancelled appointments or not.

Examining cancellations initiated by health professionals enables us to analyse the healthcare system response and to adapt to this unprecedented health crisis. An analysis of the rescheduling of cancelled appointments provides information on the ability of the healthcare system to absorb the effects of these cancellations in the months following the acute phase of the health crisis. To identify which social groups had experienced disruptions in their healthcare pathway, we studied (i) which social groups experienced cancellations by healthcare professionals during the first lockdown in France, and (ii) which social groups failed to reschedule their cancelled appointment within six months, taking into account various determinants of healthcare use, including health factors.

## Methods

### Population

EpiCov is a population-based cohort designed as a random sample of the French population aged 15 years and over at baseline. The overall objective was to understand the main epidemiological, social, and behavioural issues related to the COVID-19 epidemic in France. The sample was drawn from the national fiscal database ‘*Fichiers démographiques sur les logements et les* individus’ (FIDELI), which covers 96.4% of the population. The cohort was established in April 2020 (during the first lockdown) and included three subsequent follow-ups in November 2020 (during the second lockdown), July 2021, and November 2022. The probability sampling design and data collection have been described in detail elsewhere [16]. Data were collected using computer-assisted web interviews (CAWIs) or computer-assisted telephone interviews (CATIs). Weights from non-response models and calibration to census-derived margins were used to account for the EpiCov design and non-participation bias at baseline and each follow-up.

This study focused on participants aged 18 and older, living in metropolitan France (the area of France which is geographically in Europe), who completed the first follow up survey in November 2020. People who lived outside metropolitan France were excluded due to a different context of supply and demand for care in their area. Overall, 134 391 individuals aged 18 and older participated at baseline in April 2020 and 107 808 at the first follow-up in November 2020. Data with missing values were excluded (*n* = 4904, 4.9%). A total of 95 118 individuals were included in the study.

### Outcomes

Participants were asked if they had at least one scheduled appointment cancelled during the first lockdown (yes/no). The type of health care cancelled was then reported: general practitioner/specialist physician (including dentistry)/screening/medical exam (including radiography, CT scans, MRI, blood tests)/surgical procedure/physical therapy/others. For each type of care, respondents were asked if they cancelled their appointment on their own or if those cancellations were initiated by a health professional. Cancellation initiated by a healthcare professional was scored 1 for each individual who experienced at least one cancellation. The 0 category also included people without appointments due to lack of information. Interpretation of the results for cancellation must be done with caution: it cannot be possible to disentangle social inequalities in cancellations from social inequalities in having a scheduled appointment. Participants who experienced cancellation were asked if the appointment was rescheduled within six months. The rescheduling variable was divided into three categories: those who obtained or scheduled all the cancelled appointments, those who were unable to reschedule at least one cancelled appointment, and those who themselves decided not to reschedule at least one cancelled appointment.

#### Sociodemographic variables

We considered the following variables: age, sex, ethno-racial status, and standard of living (based on income decile *per* household consumption unit). Within the population living in France, an initial distinction is made between those who were born in metropolitan France and those born elsewhere, including both first-generation immigrants and French overseas departments (FOD) native-borns (i.e. Martinique, Guadeloupe, and Reunion Island). Among natives of metropolitan France, another distinction is made between those who do not have migrant parents (mainstream population), and those who do (second-generation immigrants). The mainstream population covers the majority of the population living in metropolitan France (78%). By contrast, immigrants, FOD native-borns and their children are minority groups. Immigrants and descendants of immigrants from Maghreb, Turkey, Africa and Asia were grouped under the term ‘racialized minorities’ [17].

#### Health variables

Health variables included self-assessed health status (very good/good/fair to very poor), number of chronic diseases (0/1/2 and more) and body mass index. We also created a binary variable indicating whether the participant reported COVID-19-like symptoms (such as cough, fever, dyspnoea, anosmia, and/or ageusia) since the beginning of the first lockdown.

#### Other covariates

The number of types of cancelled appointments was coded into four categories (0/1/2/3–7). For example, if the participant experienced a cancellation from a general practitioner and a specialist, its value was two. The three most affected regions at the time of the survey (Grand Est, Hauts-de-France, Ile-de-France) were distinguished to account for regional differences in the incidence of COVID-19 infections. We added the local potential accessibility indicator, which measures the accessibility to private general practitioners (GPs) by taking into account the level of activity of the doctor, the health needs of the population, and the health services available in the surrounding communes [18]. The higher the score, the more access to private GPs the location offers.

### Statistical analyses

Percentage of people affected by cancellations initiated by a healthcare professional was reported according to the respondents’ characteristics. In order to test the association between social factors and cancellation, adjusted prevalence ratios (PRs) and their 95% confidence interval (CI) were estimated using Poisson regressions with robust variance. Regressions were conducted in two successive steps: without and with adjustment on health variables. Association between age and cancellation may differ according to sex, partly due to the gynaecological-related consultations of women. We therefore conducted separate cancellation analyses for women and men (Supplementary Tables S1 and S2). We then performed a multinomial logistic regression among those who experienced at least one cancelled appointment to explore rescheduling (rescheduled/did not want to reschedule/failed to reschedule). Backward strategy was used for the other variables in order to fit parsimonious models. All analyses were weighted. The threshold for *P* was <0.05. Statistical analyses were performed with SAS 9.4.

### Ethics statement

The French data protection authority (*Commission Nationale de l'Informatique et des Libertés*, ref: MLD/MFI/AR205138), the ethics committee (*Comité de Protection des Personnes Sud Méditerranée* III 2020-A01191-38), and the ‘*Comité du Label de la Statistique Publique*’ approved the survey in April 2020. All participants or their legal representatives gave informed consent to participate in this study.

## Results

### Healthcare provider-initiated cancellation

Overall, 21.1% of the population (*n* = 21 511) experienced a cancellation initiated by a healthcare professional during the first lockdown (Table 1). Only 2.8% reported having cancelled at least one of their medical appointments themselves. At least 25 454 scheduled appointments were cancelled (the number of cancellations for each type of care was lacking). Most of these cancellations were for specialist care (62%), followed by physiotherapy (13%). General practice care accounted for only 3% of all cancellations. Surgical procedures and screenings accounted for 4% and 3%, respectively (Supplementary Fig. S1).

**Table 1.** Characteristics of participants according to cancellation by healthcare professional status

	Cancelled medical appointments		
	No, *n* = 73 607 (78.9%)	Yes, *n* = 21 511 (21.1%)	Total, *n* = 95 118 (100%)
Sex			
Men	35 566 (83.6)	7451 (16.4)	43 017 (46.8)
Women	38 041 (74.7)	14 060 (25.3)	52 101 (53.2)
Age (years)			
18–24	8081 (88.4)	1113 (11.6)	9194 (10.3)
25–34	9563 (81.7)	2312 (18.3)	11 875 (13.8)
35–44	12 575 (77.2)	4001 (22.8)	16 576 (15.8)
45–54	14 603 (77.5)	4580 (22.5)	19 183 (17.3)
55–64	13 658 (75.9)	4629 (24.1)	18 287 (16.6)
65–74	10 686 (76.7)	3585 (23.3)	14 271 (14.7)
75 and over	4441 (78.4)	1291 (21.6)	5732 (11.5)
Ethno-racial status			
Mainstream population	60 809 (78.5)	18 052 (21.5)	78 861 (79.5)
Born or parents born in FOD	896 (81.9)	228 (18.1)	1124 (1.3)
Non-racialized second-generation immigrants	4023 (78.5)	1170 (21.5)	5193 (5.6)
Racialized second-generation immigrants	2564 (80.3)	684 (19.7)	3248 (4.0)
Non-racialized first-generation immigrants	2371 (78.8)	704 (21.2)	3075 (4.0)
Racialized first-generation immigrants	2944 (82.7)	673 (17.3)	3617 (5.7)
Standard of living (in deciles)			
D1 (lowest)	5714 (81.5)	1461 (18.5)	7175 (7.9)
D2–D3	9306 (80.1)	2574 (19.9)	11 880 (17.1)
D4–D5	11 725 (79.7)	3237 (20.3)	14 962 (19.7)
D6–D7	15 499 (78.7)	4500 (21.3)	19 999 (21.4)
D8–D9	20 193 (77.5)	6221 (22.5)	26 414 (22.8)
D10 (highest)	11 170 (76.9)	3518 (23.1)	14 688 (11.0)
Number of chronic diseases			
0	48 109 (82.9)	10 909 (17.1)	59 018 (59.3)
1	16 495 (75.0)	6204 (25.0)	22 699 (23.9)
≥2	9003 (70.0)	4398 (30.0)	13 401 (16.8)
Perceived health status			
Fair to very bad	13 264 (72.5)	5792 (27.5)	19 056 (23.6)
Good	34 090 (78.9)	9983 (21.1)	44 073 (45.3)
Very good	26 253 (83.7)	5736 (16.3)	31 989 (31.1)
Body mass index			
Underweight	2313 (77.3)	721 (22.7)	3034 (3.2)
Normal weight	37 616 (79.2)	10 863 (20.8)	48 479 (49.1)
Overweight	23 128 (79.1)	6598 (20.9)	29 726 (31.8)
Obesity	10 550 (77.9)	3329 (22.1)	13 879 (15.8)
Region of residence according to COVID-19 infections at the time of the survey			
Least affected regions	46 146 (78.0)	14 104 (22.0)	60 250 (63.8)
Grand Est	7132 (77.0)	2312 (23.0)	9444 (8.6)
Hauts-de-France	7251 (80.4)	1951 (19.6)	9202 (9.2)
Ile-de-France	13 078 (81.9)	3144 (18.1)	16 222 (18.4)
Local potential accessibility			
Low GP accessibility	7911 (79.9)	2195 (20.1)	10 106 (10.2)
Medium GP accessibility	51 851 (78.9)	15 135 (21.1)	66 986 (70.7)
High GP accessibility	13 845 (78.4)	4181 (21.6)	18 026 (19.2)

Percentages were weighted by inverse inclusion probabilities, corrected for non-response and calibrated on the margin of census.

No gradient in prevalence of cancellations was found according to age. This finding was confirmed by multivariate analyses (Table 2). Compared with the 18- to 24-year-olds, the 35- to 44-year-olds had the highest prevalence of cancellations (PR 1.82, 95% CI 1.70–1.96) and the 75 and over had the lowest (PR 1.26, 95% CI 1.15–1.38). Stratified analyses on sex showed that men and women more experienced cancellation at different ages: 55- to 64-year-olds for men and 35- to 44-year-olds for women (Supplementary Table S1). The 75-year-olds were no longer associated with cancellation as compared to the youngest among women.

**Table 2.** Factors associated with cancellation by healthcare professionals (reference = no cancellation)

	Cancelled medical appointments (ref = no) PR (95% CI)	
	Model 1	Model 2 *+* *health variables*
Sex		
Men	1.00	1.00
Women	1.55 (1.50–1.60)	1.52 (1.47–1.57)
Age (years)		
18–24	1.00	1.00
25–34	1.55 (1.44–1.67)	1.51 (1.40–1.63)
35–44	1.94 (1.81–2.08)	1.82 (1.70–1.96)
45–54	1.91 (1.78–2.05)	1.70 (1.58–1.82)
55–64	2.00 (1.87–2.15)	1.66 (1.54–1.78)
65–74	1.90 (1.77–2.05)	1.51 (1.40–1.63)
75 and over	1.74 (1.59–1.90)	1.26 (1.15–1.38)
Ethno-racial status		
Mainstream population	1.00	1.00
Born or parents born in FOD	0.94 (0.82–1.08)	0.99 (0.93–1.06)
Non-racialized second-generation immigrants	1.00 (0.94–1.07)	0.97 (0.89–1.06)
Racialized second-generation immigrants	1.07 (0.98–1.16)	1.05 (0.97–1.15)
Non-racialized first-generation immigrants	0.97 (0.89–1.06)	0.97 (0.89–1.06)
Racialized first-generation immigrants	0.87 (0.80–0.95)	0.87 (0.80–0.95)
Standard of living (in deciles)		
D1 (lowest)	1.00	1.00
D2–D3	1.04 (0.97–1.12)	1.05 (0.97–1.13)
D4–D5	1.04 (0.97–1.12)	1.08 (1.00–1.15)
D6–D7	1.09 (1.01–1.16)	1.15 (1.07–1.23)
D8–D9	1.16 (1.09–1.24)	1.24 (1.16–1.33)
D10 (highest)	1.20 (1.12–1.29)	1.31 (1.23–1.41)
Self-assessed health		
Fair to very poor		1.25 (1.19–1.31)
Good		1.14 (1.10–1.19)
Very good		1.00
Number of chronic diseases		
0		1.00
1		1.36 (1.31–1.41)
≥2		1.62 (1.54–1.70)

Adjusted PRs and CIs were calculated considering EpiCov sampling design. PRs were also adjusted for region of residence according to COVID-19 infections at the time of the survey and local potential accessibility for models 1 and 2 and for body mass index, presenting COVID-19-like symptoms during the first lockdown for model 2.

Women experienced a higher prevalence of cancellations than men (25.3% and 16.4% respectively). On multivariate analysis, sex differences persisted (PR 1.52, 95% CI 1.47–1.57).

Ethnoracial status was not associated with cancellation experience in either univariate or multivariate analyses, except for first-generation racialized immigrants, who were less likely to experience cancellation than the mainstream population (PR 0.87, 95% CI 0.80–0.95).

There were marked differences by standard of living: the highest income decile was associated with the highest prevalence of cancellations (18.5% for the first income decile vs. 23.1% for the 10th decile). In the multivariate model, the PR increased progressively with each decile until they reached a value of 1.31 (95% CI 1.23–1.41) for the richest decile compared to the poorest decile.

Self-reported poor health and number of chronic conditions were positively associated with reporting cancellation experiences. Among those with very good health, 16.3% reported a cancellation of planned care, compared to 27.5% among those with poor health. People with no chronic conditions reported a prevalence of cancellations of 17.1%. The prevalence for those with two or more chronic conditions was 30.0%. These associations were confirmed in multivariate models. Multiple chronic conditions and poor health were associated with higher prevalence of cancellations (≥2 chronic conditions: PR 1.62, 95% CI 1.54–1.70 compared to those with no conditions, and PR 1.25, 95% CI 1.19–1.31 for those with poor health compared to those with very good health).

### Rescheduled appointments

Of those who experienced a cancellation initiated by a health professional during the first lockdown, more than 78.1% rescheduled all their appointments. Respectively 6.6% and 15.2% failed to reschedule and did not want to reschedule at least one appointment. While surgical procedure appointments were the most rescheduled (80.5%), those for physical therapy were the least (71.4%, Supplementary Fig. S2). Rescheduling rates were 79.9% and 78.1% for GPs and specialist physicians, respectively. Higher rate of failure to reschedule was found for screenings (8.0%).

There was a clear gradient in rescheduled rates according to age. The lowest and highest rates were found for younger and older people, respectively (67.1% for those aged 18–24 and 85.0% for those aged 75 and over, Table 3). This gradient effect of age was confirmed by multivariate analyses (Table 4). Those aged 18–25 years were more likely to fail to reschedule (2.67, 95% CI 1.69–4.21) and to not want to reschedule (2.58, 95% CI 1.93–3.45) than those aged 75 years and over. In all cases, the magnitude of the association decreased with age.

**Table 3.** Characteristics of participants according to rescheduling status

	Cancelled medical appointments			
	Rescheduled, *n *= 16 790 (78.1%)	Did not want to reschedule, *n* = 3349 (15.2%)	Failed to reschedule, *n* = 1372 (6.6%)	Total *n* = 21 511 (100%)
Sex				
Men	5940 (79.3)	1087 (14.6)	424 (6.1)	7451 (36.3)
Women	10 850 (77.4)	2262 (15.6)	948 (7.0)	14 060 (63.7)
Age (years)				
18–24	750 (67.1)	251 (22.7)	112 (10.2)	1113 (5.7)
25–34	1601 (69.5)	515 (22.2)	196 (8.3)	2312 (11.9)
35–44	2987 (75.0)	719 (17.7)	295 (7.4)	4001 (17.1)
45–54	3579 (77.7)	691 (14.7)	310 (7.5)	4580 (18.4)
55–64	3752 (80.6)	627 (13.4)	250 (6.0)	4629 (19.0)
65–74	3017 (84.3)	409 (11.4)	159 (4.3)	3585 (16.2)
75 and over	1104 (85.0)	137 (10.0)	50 (5.0)	1291 (11.7)
Ethno-racial status				
Mainstream population	14 177 (78.6)	2753 (14.9)	1122 (6.5)	18 052 (80.9)
Born or parents born in FOD	174 (78.5)	36 (15.0)	18 (7.5)	228 (1.1)
Non-racialized second-generation immigrants	914 (78.1)	185 (15.8)	71 (6.0)	1170 (5.7)
Racialized second-generation immigrants	484 (71.3)	135 (19.1)	65 (9.7)	684 (3.7)
Non-racialized first-generation immigrants	561 (81.8)	108 (13.8)	35 (4.4)	704 (4.1)
Racialized first-generation immigrants	480 (72.1)	132 (18.9)	61 (8.9)	673 (4.6)
Standard of living (in deciles)				
D1 (lowest)	1038 (72.8)	292 (18.4)	131 (8.9)	1461 (6.9)
D2–D3	1945 (75.5)	411 (16.0)	218 (8.5)	2574 (16.1)
D4–D5	2510 (78.1)	501 (15.0)	226 (6.8)	3237 (19.0)
D6–D7	3516 (78.6)	701 (14.9)	283 (6.5)	4500 (21.6)
D8–D9	4952 (79.9)	922 (14.4)	347 (5.7)	6221 (24.3)
D10 (highest)	2829 (80.4)	522 (14.9)	167 (4.7)	3518 (12.0)
Number of chronic diseases				
0	8376 (76.8)	1868 (17.0)	665 (6.2)	10 909 (47.9)
1	4953 (79.8)	879 (14.0)	372 (6.2)	6204 (28.3)
≥2	3461 (78.8)	602 (13.1)	335 (8.1)	4398 (23.9)
Self-assessed health				
Fair to very poor	4546 (78.6)	814 (13.6)	432 (7.8)	5792 (30.7)
Good	7851 (78.6)	1540 (15.3)	592 (6.1)	9983 (45.3)
Very good	4393 (76.6)	995 (17.3)	348 (6.1)	5736 (24.0)
COVID-19-like symptoms				
No	11 462 (80.1)	2080 (14.1)	808 (5.9)	14 350 (67.6)
Yes	5328 (74.0)	1269 (17.7)	564 (8.3)	7161 (32.4)
Number of different types of care cancelled				
1	14 387 (79.5)	2669 (14.5)	1030 (6.0)	18 086 (84.4)
2	2105 (71.5)	590 (19.3)	265 (9.3)	2960 (13.3)
3–7	298 (64.8)	90 (19.1)	77 (16.1)	465 (2.2)
Region of residence according to COVID-19 infections at the time of the survey				
Least affected regions	11 023 (78.4)	2189 (15.0)	892 (6.6)	14 104 (66.3)
Grand Est	1855 (80.1)	309 (12.9)	148 (7.0)	2312 (9.4)
Hauts-de-France	1511 (76.5)	307 (16.6)	133 (6.9)	1951 (8.5)
Ile-de-France	2401 (76.8)	544 (16.8)	199 (6.4)	3144 (15.8)
Local potential accessibility (LPA)				
Low GP accessibility	1720 (78.7)	312 (14.2)	163 (7.1)	2195 (9.7)
Medium GP accessibility	11 800 (78.1)	2380 (15.4)	955 (6.6)	15 135 (70.7)
High GP accessibility	3270 (78.0)	657 (15.3)	254 (6.7)	4181 (19.7)

Percentages were weighted by inverse inclusion probabilities, corrected for non-response and calibrated on the margin of census.

**Table 4.** Factors associated with rescheduling status (multinomial regressions, reference = rescheduled)

	Adjusted odds ratios (95% CI)	
	Failed to reschedule	Did not want to reschedule
Sex		
Men	1.00	1.00
Women	1.08 (0.92–1.26)	1.01 (0.91–1.11)
Age (years)		
18–24	2.66 (1.68–4.20)	2.61 (1.95–3.48)
25–34	2.07 (1.34–3.20)	2.45 (1.88–3.18)
35–44	1.67 (1.11–2.51)	1.81 (1.41–2.32)
45–54	1.65 (1.09–2.48)	1.48 (1.16–1.90)
55–64	1.28 (0.85–1.93)	1.35 (1.06–1.74)
65–74	0.89 (0.58–1.36)	1.13 (0.88–1.47)
75 and over	1.00	1.00
Ethno-racial status		
Mainstream population	1.00	1.00
Born or parents born in FOD	1.04 (0.61–1.78)	0.85 (0.56–1.27)
Non-racialized second-generation immigrants	0.96 (0.71–1.31)	1.15 (0.93–1.41)
Racialized second-generation immigrants	1.22 (0.88–1.68)	1.05 (0.82–1.34)
Non-racialized first-generation immigrants	0.67 (0.43–1.05)	0.95 (0.72–1.26)
Racialized first-generation immigrants	1.26 (0.89–1.79)	1.25 (0.97–1.61)
Standard of living (in deciles)		
D1 (lowest)	1.48 (1.11–1.98)	1.09 (0.89–1.34)
D2–D3	1.49 (1.14–1.95)	0.98 (0.82–1.17)
D4–D5	1.27 (0.99–1.63)	0.94 (0.79–1.10)
D6–D7	1.23 (0.97–1.56)	0.95 (0.82–1.10)
D8–D9	1.15 (0.92–1.42)	0.94 (0.82–1.07)
D10 (highest)	1.00	1.00
Number of chronic diseases		
0	1.00	1.00
1	1.04 (0.89–1.23)	0.88 (0.79–0.98)
≥2	1.49 (1.23–1.81)	0.91 (0.80–1.03)
Number of different types of care cancelled		
1	1.00	1.00
2	1.65 (1.38–1.99)	1.51 (1.34–1.71)
3–7	2.92 (2.11–4.03)	1.62 (1.19–2.22)

Adjusted odds ratios and CIs were calculated considering EpiCov sampling design. Odds ratios were also adjusted for body mass index, presenting COVID-19-like symptoms since the beginning of the first lockdown, region of residence according to COVID-19 infections at the time of the survey and local potential accessibility.

Among men, 79.3% rescheduled all their appointments vs. 77.4% of women. In multivariate analysis, sex was not associated with failing to reschedule nor with deciding not to reschedule the cancelled appointment.

Racialized minorities had the lowest rescheduling rate: 71.3 for the second-generation and 72.5% for the first vs. 78.6% for the mainstream population. In the multivariate model, non-statistically significant association was found between ethno-racial status and failing to reschedule or deciding not to reschedule the cancelled appointment.

Income was positively associated with rescheduling: rates ranged from 72.8% for the poorest decile, increasing progressively to 80.4% for the richest decile. In the multivariate model, the poorest decile was more likely to fail than the richest decile (OR 1.48, 95% CI 1.11–1.98); same result was observed for people belonging to the second and the third deciles (OR 1.49, 95% CI 1.14–1.95). The choice not to reschedule did not depend on the standard of living.

Individuals without chronic diseases have the lowest rescheduling rate as compared to their counterparts (76.8% vs. 79.8% for those with one disease and 78.8% for those with at least two). In multivariate analyses, those with at least two chronic conditions had increased odds of failure to reschedule compared with those without chronic conditions (OR 1.49, 95% CI 1.23–1.81).

## Discussion

To our knowledge, this study is the first one to provide a description of disruption in the healthcare pathway, documenting both the social groups that were the most affected by health professional-initiated cancellation, and the ones that were unable to reschedule.

One out of five people (21.1%) had at least one of their planned healthcare appointments cancelled by health professionals, mainly pertaining to specialist care (62%). Women, the richest, and those who rated their own health as poor or as having a chronic disease were the most affected by cancellations. While the vast majority of individuals who experienced cancellations had their appointments rescheduled (78.1%), the fact remains that almost 1 in 10 people (6.6%) failed to reschedule at least one appointment within six months. Their social characteristics differ from the others: the poorest and the youngest were more likely to fail to reschedule, regardless of the state of their health. We also highlighted that the elderly were not specifically affected by cancellations or by difficulties in rescheduling appointments.

Previous studies reported cancellations of medical appointments, whether initiated by healthcare professionals or by the patient. Two studies, one in the UK [10] and one in Belgium and the Netherlands [15], were conducted among adults during the early stages of the pandemic, including the lockdown period. They found cancellation rates of 15% and 26%, respectively. In comparison, our study found that a total of 23.8% experienced appointment cancellations. They were mainly initiated by healthcare professionals (21.1%), compared to those initiated by patients themselves (2.8%). Other studies, mainly in Europe [11, 12, 19, 20] and America [13, 14, 21, 22], covered a longer period, often the entire year of 2020, and were restricted to older adults. They found higher cancellation rates, ranging from 32.0% to 47.7%.

Conflicting results are reported in the literature regarding age and cancellation, possibly due to the period studied, as suggested by Wenger *et al*. [23] Some studies found that the oldest have a higher likelihood of cancelled appointments [11, 14, 23] and others found that it was the youngest who had a higher likelihood [12, 13, 21]. It could be expected that older adults would have higher cancellation rates because they have the greatest care needs, as found in an European survey on access to care [24]. In our study, people aged 75 and over were among the least affected by cancellation and were the least likely to fail to reschedule or to decide not to reschedule their cancelled appointment, as compared to the youngest. Such results suggest that health professionals tried to maintain access to care for the oldest people during the lockdown, who were more likely to die from COVID-19 in case of comorbidity. This last point would be in line with the French Public Health recommendations during the COVID-19 period which focused on the need to protect the elderly.

Consistent with the literature, we found that women were more likely to be affected by cancellations than men. We also found that they more experienced cancellations at younger ages than men, possibly explained by reproductive-related consultations. In addition to these appointments, women have a greater interest in their health and tend to be more prone to seek health care than men [8, 25]. Thus, women have on average more scheduled appointments than men, increasing their risk of cancellation. In the end, however, they were neither more likely than men to fail to reschedule an appointment nor to decide not to reschedule them.

Inconsistent results are found in the literature regarding race and cancellation. One study in the UK did not find any association [10], another one in the USA found that non-Whites experienced fewer cancellations than Whites during the first waves of COVID-19 [23], and one in the UK found the opposite, with non-Whites experiencing more cancellations, though this study did not take into account health-related factors [11]. In our study, the racialized first-generation immigrant population was less likely to experience cancellation than the mainstream population. This may be due to the fact that racialized immigrants are less used to consulting health professionals, in particular, specialists, than the mainstream population [26–28]. The oldest ones, who belong to the so-called first generation, may face an additional barrier to healthcare access due to lack of familiarity with the French healthcare system, limited language, and literacy skills. Moreover, racialized immigrants may forgo needed healthcare because of fear of interactions with public health services. However, first- and second-generation racialized minorities were neither more likely to fail to reschedule their cancelled appointments nor to decide not to reschedule them.

Finally, we found that the richest people were the most affected by cancellations. This result was to be expected, as we found that specialist care, used most by those with higher incomes [1, 2, 4], was the most affected by cancellations. Indeed, a visit to a specialist is often associated with higher costs, as specialists often charge extra fees that are only partially covered by private supplementary health insurances [29]. In the literature, most studies found that the higher the level of education or income is, the higher the rate of cancellation [12–14, 22, 23], with the exception of two studies from the UK [10, 11], of which one the authors did not adjust for health variables [11]. However, what our results also highlight is that the poorest were more likely to fail to reschedule as compared to the richest. Interestingly, the poorest were not more likely to decide not to reschedule them. Poverty is known to be a major barrier to accessing health care. The COVID-19 epidemic may have exacerbated this situation, since it led more often to loss of employment and reduced financial resources for the most socially disadvantaged people, which may have impacted their access to care [30].

Nevertheless, our study has some limitations. Detailed information on the medical appointments that were cancelled was lacking, such as the healthcare structures involved (hospital, private practice, etc.), the exact number of cancelled appointments, and the reasons for consulting. As a result, we were not able to see which were the individuals exposed to more than one cancelled appointment for each type of care, possibly resulting in underestimation of the effects. Furthermore, since cancellations concerned a period of two months, there was probably only one cancelled appointment per type of care, except for physical therapy. We also do not know to what extent these include any acute care situations, for which the consequences of not seeking help could be more severe.

Information on why people were unable to reschedule was also missing. As a consequence, we could not determine whether this was due to physician shortage or not. The survey did not identify the reason why people decided not to reschedule appointments. However, this decision does not seem to be linked to people’s state of health, as the results show.

As in all other studies but one, our data did not allow us to estimate cancellation rates only among people who had a scheduled appointment. The health inequalities in cancellations found in our study most likely reflect pre-existing inequalities in access to and utilization of health care. Performing stratified analyses on perceived health to estimate associations between people who are more likely to have similar health needs could introduce bias if there were unmeasured determinants of both need and cancellation (due to selection bias). Only one study in the USA estimated cancellation rates for adults with a medical appointment during the two months before the survey [23]. The factors associated with cancellations were the same as the others found in the literature.

We decided to carry out analyses on all individuals including those who cancelled their appointment themselves. We carried out a sensitivity analysis, excluding from the reference population those who had cancelled their appointment themselves. The results remained the same.

Finally, like any national population-based survey, our study does not cover highly vulnerable groups such as undocumented migrants and homeless people, who are particularly affected by inequalities in access to health care [31–33].

In all, despite these limitations, cancellations show the extent to which the healthcare system has failed to temporarily maintain all its functions during this unprecedented health crisis in recent decades. The fact that being rich, being healthier on average than the poor, was the most impacted by cancellation is in line with the healthcare rationing based on the most urgent treatments and procedures. However, we also found that those who reported a poorer health self-rated status and those with chronic comorbidities experienced more cancellations than others. This result suggests that the reorganization of the health system did not result in a prioritization of care based exclusively on need and health status criteria.

Analysis of the rescheduled appointments reveals the social groups that are ultimately most penalized by the restriction on access to healthcare during the crisis. While only a minority of people were unable to reschedule, our data clearly show that the poorest people were the hardest hit. Since these people are in poorer health conditions than others [29], such a result raises questions about the healthcare system’s ability to reduce social inequalities during a health crisis. Public health measures should be rapidly implemented to better protect and meet the healthcare needs of such at-risk groups.

## Supplementary Material

ckae101_Supplementary_Data

## Data Availability

The EpiCov study is available for research purpose after submission to approval of French Ethics and Regulatory Committee procedure (Comité du Secret Statistique, CESREES, and CNIL). Access procedure is available on CASD (https://www.casd.eu/). Additional information can be addressed to the corresponding author. Key points One in five people experienced health professional-initiated cancellations of medical care during the first lockdown in France. Women, the richest, and those with a chronic disease were the most affected by these cancellations. Rescheduling rate within six months was 78.1%, whereas 15.2% did not want to reschedule and 6.6% failed to reschedule. The poorest and those with at least two chronic diseases failed to reschedule more often than the rest of the population. This involuntary disruption in the healthcare pathway of the poorest might further deprive socially disadvantaged groups of the healthcare services they need.
