# Supplementary material for: Social inequalities in medical appointment cancellations and reschedulings at the onset of the COVID-19 epidemic in France
Source: Eur J Public Health. 2024 Jun 27;34(4):652–9. doi: 10.1093/eurpub/ckae101 (PMC11293806; doi:10.1093/eurpub/ckae101)
Supplement: ckae101_Supplementary_Data [file ckae101_supplementary_data.pdf]

Supplementary Data

Table S1. Prevalence of cancellations by healthcare professional according to sex.

	Cancelled medical appointments (yes)	
	Among women<br><br>n=14,060 (25.3%)	Among men<br><br>n=7,451 (16.4%)
**Age (years)**		
18-24	792 (15.8)	321 (7.4)
25-34	1,763 (24.6)	549 (11.1)
35-44	2,846 (29.5)	1,155 (15.4)
45-54	2,974 (27.3)	1,605 (17.4)
55-64	2,916 (27.7)	1,713 (20.2)
65-74	2,076 (25.4)	1,509 (20.7)
75 and over	692 (22.4)	599 (20.4)
**Ethno-racial status**		
Mainstream population	11,746 (25.7)	6,306 (16.7)
Born or parents born in FOD	153 (21.4)	75 (14.5)
Non-racialised second-generation immigrants	780 (26.7)	390 (15.6)
Racialised second-generation immigrants	488 (25.4)	196 (12.8)
Non-racialised first-generation immigrants	474 (23.8)	230 (17.6)
Racialised first-generation immigrants	419 (20.2)	254 (14.3)
**Standard of living (in deciles)**		
D1 (lowest)	1,023 (22.3)	438 (13.8)
D2-D3	1,810 (23.8)	764 (14.8)
D4-D5	2,198 (24.2)	1,039 (15.7)
D6-D7	2,964 (25.5)	1,536 (16.7)
D8-D9	3,901 (27.4)	2,320 (17.5)
D10 (highest)	2,164 (27.8)	1,354 (18.3)
**Number of chronic diseases**		
0	7,417 (21.7)	3,492 (12.0)
1	3,960 (29.3)	2,244 (20.1)
≥2	2,683 (31.7)	1,715 (27.8)
**Perceived health status**		
Fair to very bad	3,700 (30.1)	2,092 (24.0)
Good	6,537 (25.4)	3,446 (16.3)
Very good	3,823 (21.1)	1,913 (11.5)
**Body Mass Index**		
Underweight	630 (26.1)	91 (13.6)
Normal weight	7,789 (25.3)	3,074 (14.9)
Overweight	3,567 (25.4)	3,031 (17.4)
Obesity	2,074 (25.2)	1,255 (18.5)

FOD: French overseas departments; *GP*: General Practitioner.
Percentages were weighted by inverse inclusion probabilities, corrected for non-response and calibrated on the margin of census.

Table S2. Factors associated with cancellation by healthcare professionals according to sex (reference=no cancellation).

	Cancelled medical appointments (ref=no) PR (95% CI)			
	Women		Men	
	Model 1	Model 2 *+ health variables*	Model 1	Model 2 *+ health variables*
**Age (years)**				
18-24	1.00	1.00	1.00	1.00
25-34	1.56 (1.43-1.70)	1.53 (1.40-1.66)	1.51 (1.30-1.75)	1.46 (1.26-1.70)
35-44	1.87 (1.72-2.03)	1.78 (1.64-1.93)	2.07 (1.82-2.37)	1.92 (1.68-2.20)
45-54	1.72 (1.58-1.86)	1.56 (1.43-1.69)	2.34 (2.06-2.67)	2.01 (1.76-2.30)
55-64	1.70 (1.56-1.85)	1.47 (1.35-1.60)	2.67 (2.35-3.04)	2.06 (1.80-2.36)
65-74	1.55 (1.42-1.69)	1.29 (1.18-1.42)	2.72 (2.39-3.10)	1.96 (1.71-2.25)
75 and over	1.37 (1.23-1.53)	1.06 (0.94-1.19)	2.68 (2.30-3.11)	1.77 (1.51-2.06)
**Ethno-racial status**				
Mainstream population	1.00	1.00	1.00	1.00
Born or parents born in FOD	0.89 (0.76-1.06)	0.88 (0.75-1.04)	0.94 (0.82-1.08)	0.99 (0.77-1.27)
Non-racialised second-generation immigrants	1.06 (0.97-1.15)	1.05 (0.97-1.14)	0.91 (0.81-1.02)	0.90 (0.80-1.01)
Racialised second-generation immigrants	1.09 (0.99-1.20)	1.07 (0.98-1.18)	1.00 (0.85-1.18)	0.99 (0.84-1.16)
Non-racialised first-generation immigrants	0.94 (0.85-1.05)	0.95 (0.85-1.05)	1.02 (0.88-1.19)	1.03 (0.88-1.19)
Racialised first-generation immigrants	0.82 (0.73-0.91)	0.81 (0.73-0.91)	0.93 (0.80-1.08)	0.95 (0.82-1.10)
**Standard of living (in deciles)**				
D1 (lowest)	1.00	1.00	1.00	1.00
D2-D3	1.05 (0.96-1.14)	1.06 (0.97-1.15)	1.03 (0.90-1.18)	1.05 (0.92-1.20)
D4-D5	1.04 (0.96-1.14)	1.06 (0.98-1.16)	1.05 (0.92-1.20)	1.11 (0.97-1.26)
D6-D7	1.09 (1.01-1.18)	1.14 (1.05-1.23)	1.09 (0.96-1.23)	1.16 (1.03-1.32)
D8-D9	1.18 (1.09-1.28)	1.25 (1.15-1.35)	1.13 (1.00-1.27)	1.24 (1.10-1.40)
D10 (highest)	1.22 (1.12-1.32)	1.31 (1.20-1.42)	1.17 (1.03-1.33)	1.32 (1.16-1.49)
**Self-assessed health**				
Fair to very poor		1.23 (1.16-1.31)		1.28 (1.17-1.40)
Good		1.14 (1.09-1.19)		1.13 (1.06-1.21)
Very good		1.00		1.00
**Number of chronic diseases**				
0		1.00		1.00
1		1.30 (1.25-1.36)		1.43 (1.34-1.53)
≥2		1.47 (1.39-1.56)		1.88 (1.73-2.04)

CI: Confidence Interval; *FOD*: French overseas departments.

Adjusted prevalence ratios (PR) and confidence intervals (CI) were calculated considering EpiCov sampling design. Prevalence ratios were also adjusted for region of residence according to Covid-19 infections at the time of the survey and Local Potential Accessibility for model 1 and 2 and for body mass index, presenting Covid-19-like symptoms during the first lockdown for model 2.

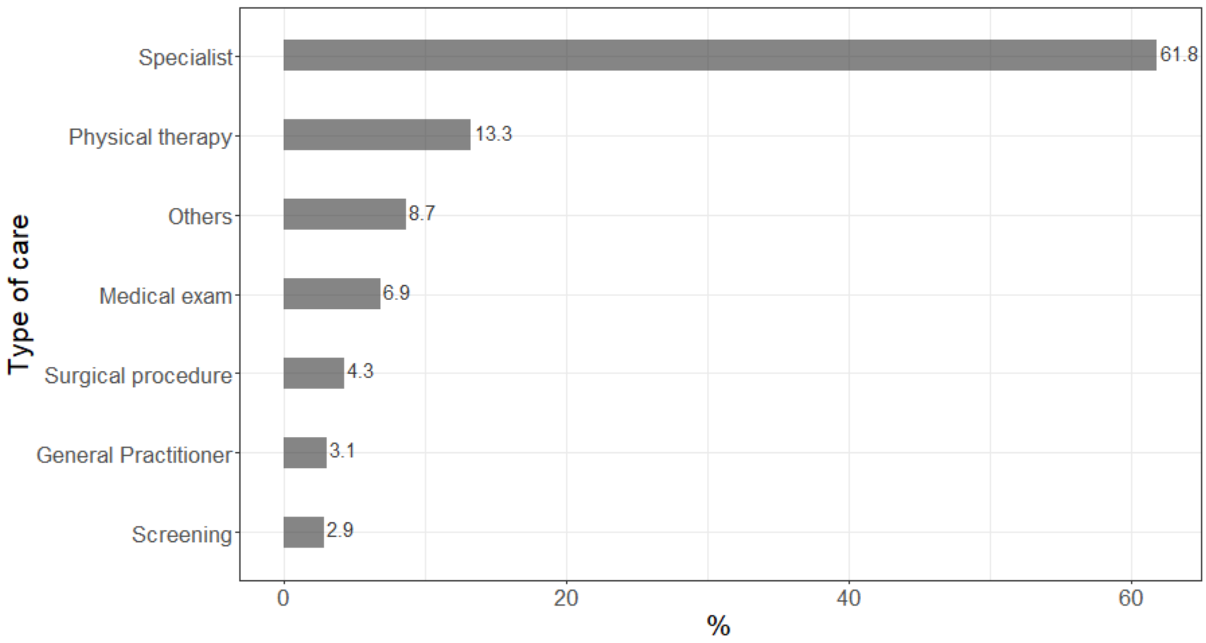

Figure S1. Distribution of the cancelled appointments by type of care.

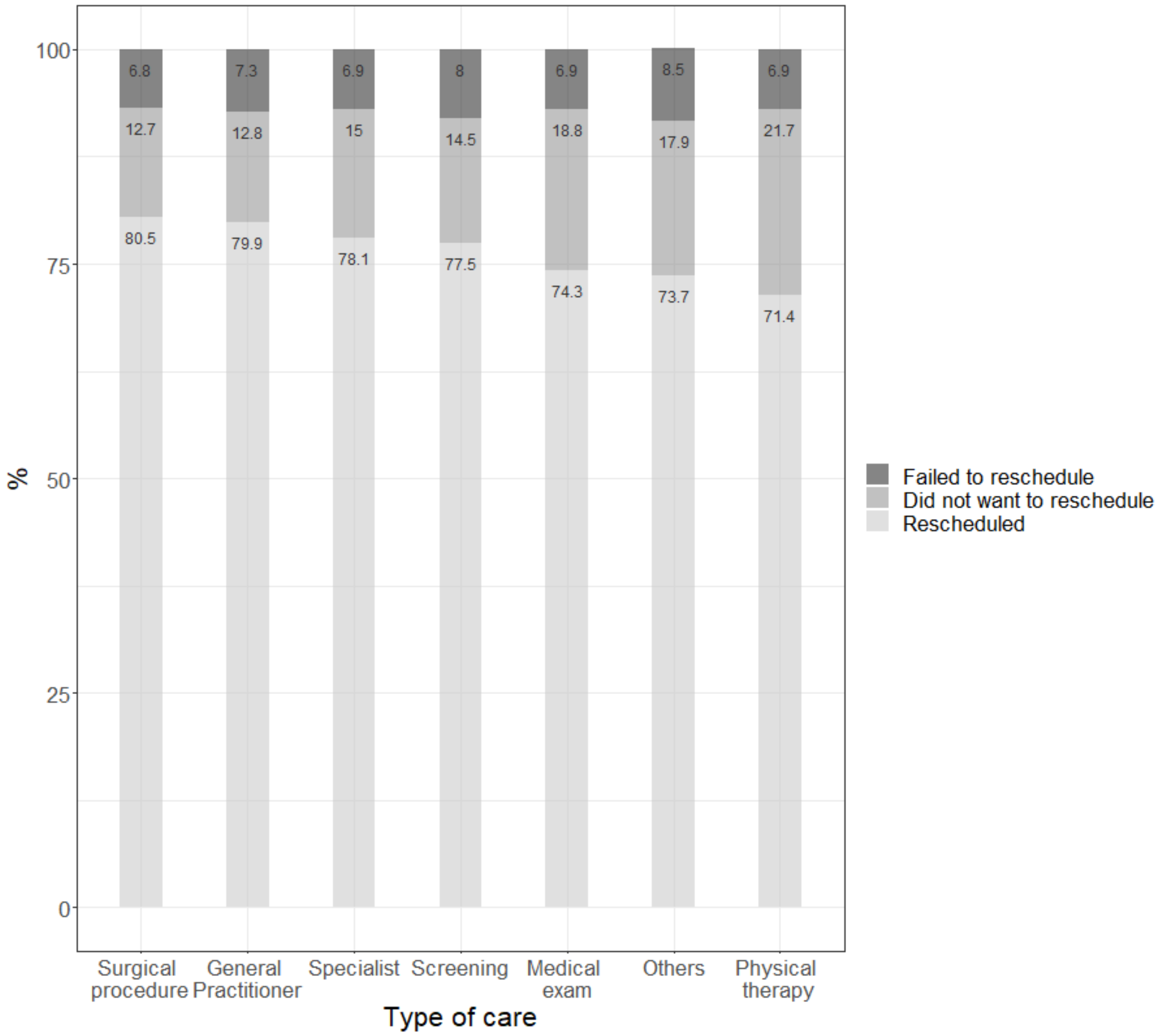

Figure S2. Rescheduled cancelled appointments by type of care.
